# *Akkermansia muciniphila* alleviates metabolic disorders through gut microbiota-mediated tryptophan regulation

**DOI:** 10.1186/s13568-025-01986-3

**Published:** 2025-11-22

**Authors:** Fengli Zhang, Jiajun Tian, Ji-Tong Li, Tengyuan Zhang, Xiao Wang

**Affiliations:** 1 Department of Aquaculture, Zhejiang Ocean University, Zhoushan, 316000 Zhejiang China; 2 Health Science Center, Ningbo University, Ningbo, 315211 Zhejiang China

**Keywords:** *Akkermansia muciniphila*, Hepatic steatosis, Intestinal barrier, Intestinal microbiota, Tryptophan metabolism

## Abstract

High-fat diet (HFD) consumption poses a significant public health threat due to its strong association with obesity, hyperlipidemia, cardiovascular diseases, and metabolic syndrome. While *Akkermansia muciniphila* has emerged as a promising probiotic for ameliorating metabolic disorders, the underlying mechanisms-specifically its interplay with intestinal tryptophan metabolism-remain poorly understood. Here, we demonstrate that *A. muciniphila* supplementation mitigates HFD-induced metabolic dysfunction in zebrafish by orchestrating a gut microbiota-tryptophan metabolite-AhR signaling axis. Firstly, *A. muciniphila* treatment effectively reduced the final weight of zebrafish, partially mitigated weight gain caused by HFD, and improved survival rate. In addition, *A. muciniphila* treatment reduced zebrafish adiposity, hepatic steatosis, and endotoxemia while enhancing the antioxidant capacity of the liver. We further found that *A. muciniphila* exerted anti-apoptotic effects, improved chronic low-grade inflammatory responses, enhanced antioxidant capacity, and repaired intestinal barriers induced by HFD. These effects were partially abolished by antibiotic pretreatment, confirming microbiota dependency. Subsequent 16S *r*RNA sequencing analysis revealed that *A. muciniphila* supplementation reshaped gut microbiota, enriching *Staphylococcus* and *Vibrionacea*e, while depleting *Acinetobacter*, *Perlucidica*, and *Massilia*. In addition to reducing lipid synthesis, metabolomic profiled the substantial changes in microbiota-regulated tryptophan metabolism, including increased levels of indole-3-lactic acid, indole-3-acetaldehyde, and 5-hydroxyindole acetic acid in Amuc group. These metabolites activated aryl hydrocarbon receptor (AhR), upregulating downstream *IL-22* and improving intestinal integrity and chronic inflammatory response. Pathway analysis and enzyme gene expression detection indicated that *A. muciniphila* may inhibit the KP pathway while promoting the microbiota-dependent tryptophan metabolic pathway. This study establishes a novel link by which *A. muciniphila* exerts protective effects against HFD-induced metabolic syndrome through microbiota-mediated enhancement of tryptophan metabolism.

**Supplementary Information:**

The online version contains supplementary material available at 10.1186/s13568-025-01986-3.

## Introduction

Obesity prevalence has doubled in over 70 countries since 1980, and it has been steadily rising in most countries (Organization WH 2021). Epidemiological studies show that obesity and overweight are often accompanied by cardiovascular diseases (CVD), diabetes mellitus, metabolic dysfunction-associated steatotic liver disease (MASLD), chronic kidney disease (CKD), multiple cancers, etc. (Gaiţă and Moşteoru 2017; Stasi et al. 2022). These illnesses rank among the top 10 global causes of death. In recent decades, besides genetic factors contributing to obesity, the imbalance between energy intake and energy expenditure caused by overnutrition is the dominant factor (Golay and Bobbioni 1997). Systemic persistent low-grade inflammation and a substantial accumulation of body fat are characteristics of obesity (Esser et al. 2014). The gut microbiota is a major mediator in the relationship between the development of obesity and the consumption of high-fat diets (HFDs), according to a number of studies (Liu et al. 2020).

Gut bacteria and host must be able to talk to each other in order to keep host metabolism and immunity in balance (Marchesi et al. 2016). Current research suggests that a HFD is highly correlated with the dysbiotic gut microbial structure, this change occurs within a day. Obese individuals have less microbial diversity, accompanied by large variation in core microorganisms (Turnbaugh and Gordon 2009). Meanwhile, prior studies noted that HFD-fed mice have abundant gut Pseudomonadota, whose increased abundance may indicate dysbiosis and higher disease risk (Zhu et al. 2023; Duan et al. 2024). Specific intestinal bacteria taxa (*Lachnospiraceae*, *Desulfovibrio*) have been suggested to exacerbate HFD-induced metabolic disorders and obesity, while others are negatively associated with these diseases (Yoon et al. 2021; Takeuchi et al. 2023; Qi et al. 2024; Aron-Wisnewsky et al. 2020). Gut microbiota plays a major role in the development and progression of fatty liver disease. The transfer of microbiota from obese animals to germ-free animals leads to increased body fat accumulation, while the germ-free animal model is safeguarded against obesity's effects (Turnbaugh et al. 2006). Fatty liver disease induced by HFD can be prevented by repairing the disruption of microbiota (Porras et al. 2017; Ding et al. 2020). The gut microbiota intervention has demonstrated significant therapeutic advantages for the clinical treatment of MASLD (Malesza et al. 2021; Wu et al. 2024). *Bacteroides vulgaris* reduces fat absorption and inhibits 5-HT production to mitigate the effects of HFD-induced obesity (Wen et al. 2024). By triggering the liver receptor acetate/GPR43/IL-6/JAK1/STAT3 axis, *Bifidobacterium pseudolongum* can alter the gut microbiota's composition and prevent the growth of MASLD -HCC (Song et al. 2023). *Bacteroides uniformis* has been identified to improve the progression of MAFLD through novel bile acid 3-sucCA, and its beneficial effects depend on the presence of gut microbiota (Nie et al. 2024). Furthermore, nicotinamide, a metabolite of *B. uniformis*, promotes the abundance of *Lactobacillus Johnsonii* through bacterial cross-feeding. Its release of indole-3-lactic acid (ILA) is a key bioactive substance that activates AhR (Zhang et al. 2024). The direct or indirect effects of potential probiotics render them desirable therapeutic target for HFD-induced metabolic syndrome.

The gut microbiota and its metabolites play a major role in the physiological metabolism of the host. The three most researched intestinal bacterial metabolites are tryptophan metabolites, bile acids generated, and short-chain fatty acids (SCFAs). Among them, the intestinal microbiota can metabolize 4–6% of tryptophan through the indole pathway to produce indole and its derivatives (IDs) (Agus et al. 2018). By activating AhR, IDs, which are AhR's natural ligands, have been demonstrated to enhance the intestinal barrier (Liu et al. 2023). It has been reported that HFD leads to the consumption of tryptophan metabolites that depend on microbial communities (Krishnan et al. 2018). The impairment of AhR activity and HFD-induced metabolic syndrome are significantly positively correlated (Natividad et al. 2018). Supplementing with AhR agonists and ligands can lead to improvements in metabolic disorders, including inflammatory responses and hepatic steatosis (Postal et al. 2020). *Akkermansia muciniphila* is a symbiotic bacterium in the mucous layer, as a next-generation candidate probiotic, it has garnered a lot of attention due to its role in treating obesity, Type 2 Diabetes, and oxidative stress of aging (Everard et al. 2013; Estefanía et al. 2021). Yin et al. (2021) found a reciprocal interaction between tryptophan and *A. muciniphila* in vitro (Yin et al. 2021). It can metabolize tryptophan to indole, IAA, indole-3-carboxaldehyde, and ILA, these IDs, in turn, promote its growth. According to a preliminary investigation, *A. muciniphila* relieves colitis in mice by blocking the kynurenine pathway's (KP) activation for tryptophan metabolism (Gu et al. 2021). Further, it may inhibit the progression of CRC by downregulating AhR (Zhang et al. 2023a, b). Shi et al. (2021) linked AhR ligands and *A. muciniphila* abundance to impeding saccharin/sucralose-induced MASLD (Shi et al. 2021). However, there has been no evidence on the direct intervention of *A. muciniphila* in regulating microbial-tryptophan metabolism pathway to improve metabolic syndrome induced by HFD. Therefore, further clarification is needed on whether *A. muciniphila* improves metabolic syndrome by regulating gut microbiota composition, and restoring HFD-induced depletion of the tryptophan metabolites in indole pathway.

As a model organism, the zebrafish shares similar mechanisms for regulating metabolism with mammals and has been extensively applied in almost all fields of human diseases, including lipid metabolism (Clifton et al. 2010). In the current study, we determined whether *A. muciniphila* could regulate hepatic metabolic dysfunction and intestinal inflammation in HFD-induced zebrafish. And we further used antibiotic intervention was to evaluate whether this prebiotic effect is mediated by intestinal microbiome. Moreover, we utilized omics techniques to investigate the impact of *A. muciniphila* on intestine metabolism profile, and utilized correlation analysis to explain how gut microbiota alleviates metabolic disorders and immune response caused by HFD through tryptophan metabolism. Moreover, after *A. muciniphila* intervention, the three major tryptophan metabolic pathways, as well as the expression of AhR and its downstream genes, were evaluated. This study preliminarily clarifies that *A. muciniphila* corrects HFD-induced metabolic abnormalities through gut microbiota-dependent regulation of tryptophan metabolism.

## Methods

### Fish husbandry

All experiments and fish care procedures were approved by the Experimental Animal Welfare Ethics Committee of Zhejiang Ocean University (Assurance No. 2024050). Adult zebrafish (1-month-old) for ***Experiment 1*** and ***Experiment 2 ***were cultured in 5L tanks in a recirculating system (n = 6 tanks/diet treatment, 20 fish/tank). Feeding conditions were as follows: temperature 27 ± 1 °C; inlet rate 120 L/h; dissolved oxygen (DO) > 6.0 mg/L; nitrogen < 0.02mg /L; nitrite < 0.05 mg/L; pH 7 ± 0.5.

### Feeding experiments and diets

#### Experiment 1

In this experiment, *A. muciniphila* (ATCC BAA-835, BioSci, Hangzhou, China) was supplemented to investigate its role in improving HFD-induced metabolic syndrome such as hepatic steatosis, intestinal homeostasis disorders of zebrafish. The crude lipid content of the basal diet (LFD) was 6%, and the high-fat diet (HFD) was used to induce fatty liver model with lipid content of 16%. On the basis of the HFD, 10^7^ cfu/g, 10^8^ cfu/g, 10^9^ cfu/g of *A. muciniphila* were added respectively (Amuc) (Supplementary Table 1). The experiment lasted for 4 weeks, the suitable effective dosage of *A. muciniphila* was determined firstly. Blood was extracted by tail amputation, placed at 4 °C for 30 min, then centrifuged (2000 g, 4 °C, 2 min) subsequently and the serum was obtained for detection. After the liver and intestinal tissue were taken out, then rinsed with PBS for detection of biochemical parameters. After the last feeding for 12 h, intact liver and intestine were taken carefully and fixed with 4% paraformaldehyde immediately for morphological observation. Liver and intestinal samples were transferred into 1.5 ml RNA-free tube filled with Trizol reagents, for detecting mRNA expression level. Intestinal content (12 tails/sample) was submitted to the detection of Untargeted metabolomics and 16 s *r*RNA high-throughput sequencing.

#### Experiment 2

In order to find out if the gut microbiota mediates the impact of *A. muciniphila* on HFD-induced fatty liver in zebrafish, antibiotics (0.25% polymyxin and 0.33% neomycin) were added to each of the three diets in ***Experiment 1***. LFD and HFD with antibiotics served as negative and positive controls, respectively. The feeding period and conditions were consistent with those described in ***Experiment 1***. After the completion of intervention culture, the body weight of the antibiotic-treated (Abx) zebrafish was measured, as were the triacylglycerol (TAG) levels in the liver and muscles. Moreover, the morphology of the intestines and liver was detected by hematoxylin–eosin (H&E) staining.

### Hematoxylin–eosin (H&E) staining, Alcian blue-PAS (AB-PAS)

Liver and intestinal tissues from adult zebrafish in Experiments 1 and 2 were extracted, washed twice with PBS, and fixed in 4% paraformaldehyde for H&E and AB-PAS staining, as described by Zhang et al (2019). Intestinal tissues were embedded in paraffin and prepared for AB-PAS staining. Subsequently, the sections were dehydrated and made transparent and rinsed with PBS buffer first. Then it was stained with alcian blue solution (sigma, America), followed by three wash steps every 5 min with distilled water. Then the sample as immersed in schiff reagent and re-stained with hematoxylin. The slice was differentiated with acid solution, reversed blue with Scott's blue solution, and dehydrated with ethanol. The representative image of each group was microscopically observed using a microscope (Leica DMIL-LED, Germany).

### Biochemical parameters analysis

The collection of zebrafish serum samples in ***Experiment 1*** was used for detecting biochemical indicators. Alanine transaminase (ALT) and aspartate aminotransferase (AST) were measured with commercial kits (Nanjing Jiancheng Bioengineering Inc., China) following the manufacturer’s instructions. The diluted serum was successively added with matrix solution, dinitrophenylhydrazine, and sodium hydroxide, and OD_510_ was determined after the reaction was completed. The standard curve was fitted and the enzyme activity of each sample was calculated, expressed as U/L.

Zebrafish liver from four fish was pooled as a sample (about 10mg) for TAG determination in ***Experiment 1*** and ***Experiment 2***. The method was referred to as previously described with minor modifications (Zhang et al. 2019). The TAG content was normalized by the protein (mg) of liver and muscle, and expressed as ug/mg prot. The total cholesterol (T-CHO) (Nanjing Jiancheng Bioengi-neering Inc., China) content of liver was also measured. The working liquid containing cholesterol esterase, cholesterol oxidase and peroxidase was pre-prepared and mixed with the sample (100:1) at 37 °C for 10 min, and the OD_500_ was determined with the microplate reader. Hepatic Malondialdehyde (MDA) was measured by Lipid Peroxidation MDA Assay Kit (Beyotime, China). The homogenate of the liver sample was mixed with TBA diluent, TBA storage solution and the mixture of antioxidants at a ratio of 1:2, heated at 100 °C for 15 min, centrifuged at 1000 g for 10 min, and determined at 532 nm with a microplate reader.

Serum endotoxin levels were determined using the ToxinSensor™ Chromogenic LAL Endotoxin Assay Kit (GenScript) following the manufacturer's protocol, with results reported in endotoxin units per milliliter (EU/ml). Intestinal diamine oxidase (DAO) activity was assessed via a commercially available ultraviolet spectrophotometric assay (Grace Biotechnology Co.,Ltd). The intestinal tissue was weighed and homogenated in PBS at 4 °C. The determination method of DAO activity follows the manufacturer's instruction, which is based on its catalytic formation of aldehyde and ammonia by diamine, and the formation of NADH by ammonia under the action of glutamate dehydrogenase. The change of NADH at OD_340_ was determined by ultraviolet light method to calculate the activity of DAO. The oxidation of 1 nmol NADH per milligram of tissue per minute is defined as a unit of enzyme activity. Intestine and liver samples of homogenates were centrifuged and the supernatant was used to evaluate Total antioxidant capacity (T-AOC) activity (Nanjing Jiancheng Bioengi-neering Inc., China). Peroxidase and ABTS working liquid were added to the reaction system successively, and the reaction was carried out at room temperature for 6 min. The wavelength of 410 nm was measured, and the standard curve of Trolox was used to calculate the antioxidant capacity of each sample (mmol/g). Liver superoxide dismutase (SOD) activity was further tested according to the instructions of the kit (Beyotime Biotechnology).

### Intestine microbiota analysis

Within 4–6 h of the last feeding after the 4-week, intestinal contents were collected. And the genomic DNA was extracted with Fast DNA spin kit (MP, Biomedicls). The quality and concentration of DNA extraction were assessed prior to sequencing. The V3-V4 region of 16S *r*RNA was amplified with U341F and U806R, and amplification results were delivered to Illumina HiSeq platform (Majorbio, China). The paired-end sequences of each sample were deposited with NCBI SRA and are available under accession no. PRJNA1094899. The raw data was processed by concatenation and quality control, denoised with DADA2, the representative sequences were output and then grouped into ASV (Amplicon Sequence Variants). Mothur 1.30.2 was used for α-diversity (ACE, Chao, Shannon, Simpson indices). QIIME 1.9.1 assessed β-diversity via PCoA based on Bray Curtis distance to test microbial community similarity across groups. Bar charts visualized *A. muciniphila*'s effects on intestinal community relative abundance at phylum and genus levels using taxonomic analysis results. Phylogenetic Investigation of Communities by Reconstruction of Unobserved States (PICRUSt2) used the representative sequences from ASV to predict the metagenomic function. Furthermore, based on the KEGG pathway in the PICRUSt2 module at the level 3 classification, the gut microbiota was predicted to specifically compare the differences in tryptophan metabolism among different groups.

### Untargeted metabolomics of intestine

After 4 h of feeding, the intestine of zebrafish (5 fish pooled as a sample, 6 replicates per group) was collected for nontargeted metabolomics analysis. The pre-treatment process of the sample was performed as follows: 25 ± 5 mg sample was weighed and homogenized with 200 μl extraction solution (methanol: water = 4:1). The supernatant was obtained by centrifugation (13,000 g, 4 °C, 15 min) for LC–MS (UHPLC-Q Exactive HF-X) detection. The chromatographic column was ACQUITY UPLC HSS T3 (100mm × 2.1mmi.d., 1.8μm; Waters, Milford, USA), mobile phase A was 95% water + 5% acetonitrile (containing 0.1% formic acid), and mobile phase B was 47.5% acetonitrile + 47.5% isopropanol + 5% water (containing 0.1% formic acid), with an injection volume of 3 μl, and a column temperature of 40 °C. The sample was ionized by electrospray, and the mass spectrum signals were collected by positive and negative ion scanning modes respectively. Quality control samples were used to assess the stability of the entire testing process. And the raw data was imported into the metabolomics processing software ProgenesisQI v3.0 (Waters Corporation, Milford, USA) for raw data matrix analysis and metabolites identification. VIP values were calculated, with metabolites having VIP ≥ 1 considered significant. Differential metabolites (DMs) were screened by VIP ≥ 1 and *p* < 0.05. Fold change indicated DM differences between HFD vs LFD and Amuc vs HFD. The volcano plot was used to visualize the changes in differential metabolites between the Amuc vs HFD and HFD vs LFD groups (Fold Change ≥ 1, *p*-value < 0.05). A supervised PLS-DA (partial least-squares discriminant analysis, PLS-DA) was performed on MajorBio Cloud to reveal global metabolic changes among the three dietary groups. Subsequently, the differential metabolites of different groups were mainly mapped to which KEGG pathways they belonged to, and pathway enrichment analysis was performed between different groups. The Heatmap and clustering tree were constructed to analyze the global differential metabolites among the different groups.

### Correlation analysis between gut microbiota and differential metabolites

The Spearman correlation analysis was utilized to explore the correlation between gut microbiota and differential metabolites. Metabolites, as subsets of environmental factors, were analyzed alongside bacterial genera using the Majorbio cloud platform (https://cloud.majorbio.com). All statistical analyses employed two-sided tests with *p* < 0.05 as the significance threshold. Correlations were assessed and adjusted for multiple comparisons via false discovery rate (FDR).

### RNA extraction, and *q*RT-PCR analysis

Total RNA of zebrafish intestinal and liver tissue was extracted with TRIzol reagent (Cowin, China). The samples were homogenized and centrifuged at 13,000 g and 4 °C for 15 min. The supernatant was mixed with 200 μl chloroform and vortexed, then centrifuged as above. The supernatant was transferred to new RNA-free EP tube, 400 μl isopropyl alcohol was added and gently mixed, and placed at − 20 °C for 1 h. The RNA was reverse-transcribed into cDNA with one-step method using FastKing RT reagent kit (TIANGEN, China). Subsequent *q*RT-PCR analysis using cDNA as a template was carried out by SYBR Green FsatReal RT-PCR premix (TIANGEN, China). The reaction procedure followed the method described previously, and the annealing temperature was modified according to each specific primer (Supplementary Table 2). Using Rps11 as the reference gene, the results were normalized, and the relative expression of genes at mRNA level was analyzed by 2^−ΔΔCT^ method. The LFD group served as the control, with its value set to 1, and the other two groups are presented as fold of control.

### Statistics analysis

Statistical data reported here are shown as mean ± SEM. All statistical analyses were performed by GraphPad Prism 8.0.1. Comparisons between the two groups were performed using Student’s *t* -tests (and nonparametric tests). Tukey's post hoc tests were conducted after a one-way analysis of variance (ANOVA) to evaluate multiple sample differences. The data of ***Experiment 1*** and ***Experiment 2*** were compared with the HFD or corresponding antibiotic intervention group, and the significance was marked directly above the corresponding group column (* represents *p* < 0.05, **represents *p* < 0.01, ***represents *p* < 0.001).

## Results

### *Akkermansia muciniphila* altered growth performance and alleviated HFD-induced hepatic steatosis

Firstly, among different *A. muciniphila* doses tested, 10^8^ cfu/g significantly reduced hepatic TAG content in ***Experiment 1*** (*p* < 0.05; Supplementary Fig. 1). Our growth performance study showed that supplementing HFD with *A. muciniphila* did not affect zebrafish survival rate (SR), whereas HFD alone significantly decreased SR (*p* < 0.05). Meanwhile, *A. muciniphila* supplementation had no impact on zebrafish weight gain rate (WGR) or specific growth rate (SGR) (Supplementary Table 3). Based on these results, this dose was selected for subsequent experiments following a comprehensive evaluation of its effects on growth performance.

**Fig. 1:**
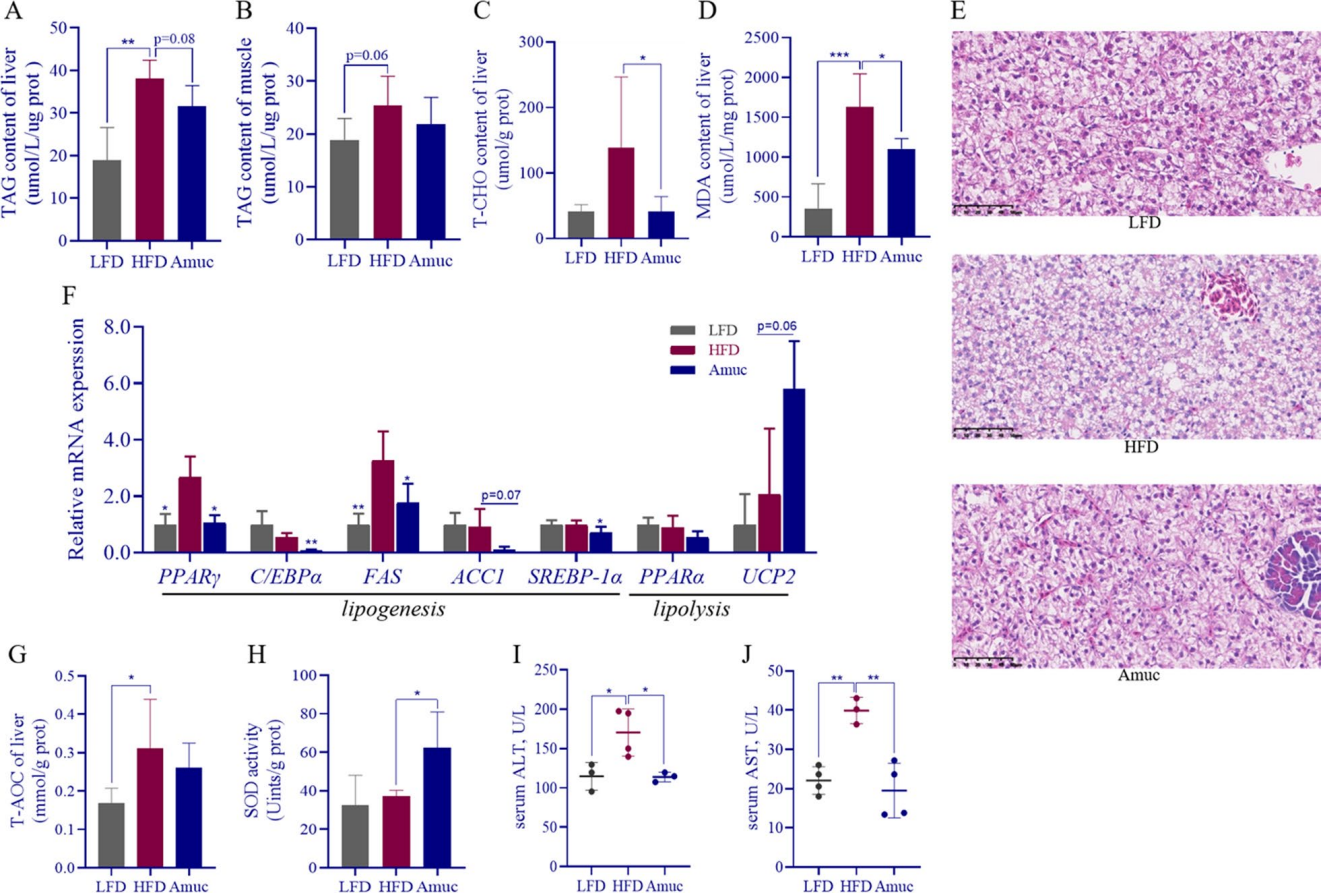
*Akkermansia muciniphila* alleviates HFD-induced hepatic lipid metabolism disorders and enhances antioxidant capacity in zebrafish. (n = 3–6). **A**–**D** Liver TAG, muscle TAG, liver T-CHO, liver MDA content of zebrafish in *Experiment 1*. **E** Representative image of H&E staining of liver given three different dietary different experimental diets (scale bar = 50 μm). **F** Lipid metabolism-related genes' relative mRNA expression of liver.** G**,** H** Liver T-AOC, SOD activity of zebrafish. **I** Serum ALT and** J** AST levels of zebrafish. Data are presented as mean ± SEM. Student’s *t*-test was used for statistical analysis, with *p < 0.05, **p < 0.01, and ***p < 0.001

We further investigated the effect of *A. muciniphila* addition on HFD-induced lipid metabolism disorders and liver injury. This experiment showed that HFD significantly increased hepatic fat accumulation in zebrafish (*p* < 0.001), *A. muciniphila* supplementation exhibited a distinct tendency to reduce (*p* = 0.08; Fig. 1A). No significant effect on muscle TAG content was observed (Fig. 1B). *Akkermansia muciniphila* could significantly reduce the content of T-CHO in zebrafish liver (*p* < 0.05; Fig. 1C). HFD significantly increased oxidative stress of zebrafish liver manifested as higher level of MDA (*p* < 0.01), and the addition of *A. muciniphila* can effectively reverse the negative effects (*p* < 0.05; Fig. 1D). Liver H&E staining confirmed *A. muciniphila*'s role in improving hepatic steatosis, as its supplementation notably reduced HFD-induced liver fat droplet accumulation and enlargement (Fig. 1E).

To further investigate how *A. muciniphila* regulates hepatic lipid metabolism, mRNA expression of lipolysis- and lipogenesis-related genes was assessed. Consistently, *A. muciniphila* significantly downregulated peroxisome proliferator-activated receptor γ (*PPARγ*) (*p* < 0.05), CCAAT enhancer binding proteins (*C/EBPα*) (*p* < 0.01), fatty acid synthetase (*FAS*) (*p* < 0.05), Sterol Regulatory Element-Binding Protein 1α (*SREBP-1α*) (*p* < 0.05), and while Uncoupling protein 2 (*UCP2*) expression showed an upward trend (*p* = 0.06; Fig. 1F). However, it had no significant effect on acetyl-CoA carboxylase1 (ACC1) or peroxisome proliferator-activated receptor α (*PPARα*). HFD impairs hepatic antioxidant capacity, which is manifested by a significant compensatory increase in T-AOC (*p* < 0.05, Fig. 1G). *A. muciniphila* supplementation had no significant effect on T-AOC, but significantly increased SOD activity (*p* < 0.05, Fig. 1H). And *A. muciniphila* can effectively restore HFD-induced liver injury, manifested by a significant decrease in serum ALT and AST levels (*p* < 0.05, *p* < 0.01; Fig. 1I, J). The above results indicate that *A. muciniphila* could improve HFD-induced hepatic steatosis via inhibiting lipid synthesis and promoting lipolysis by regulating key genes. Moreover, it can boost antioxidant capacity while alleviating liver oxidative stress and damage.

### *Akkermansia muciniphila* mitigates HFD-induced gut injury and improves barrier function

**Fig. 2:**
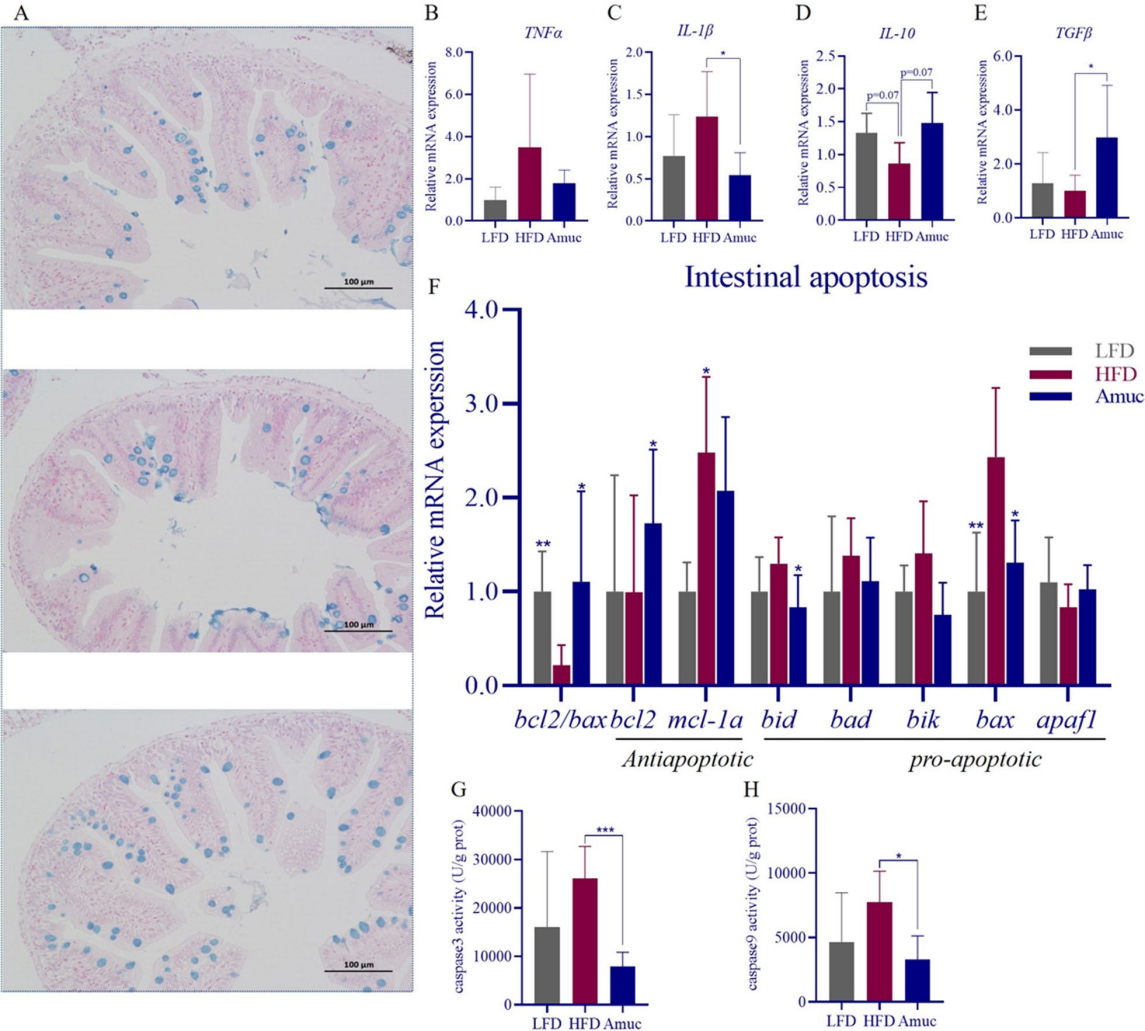
*Akkermansia muciniphila* alleviates HFD-induced intestinal inflammation and apoptosis, and maintains normal intestinal morphology. **A** Results of AB-PAS staining of the intestines of zebrafish fed different diets. Scale bar = 100 μm. **B**–**E** mRNA expression of genes (*TNFα*, *IL-1β*, IL-10, TGF*β*) related to intestinal inflammation of zebrafish. (n = 4–6) **F** Expression of intestinal apoptosis related genes (*bcl2/bax*, *bcl2*, *mcl-1α*, *bid*, *bad*, *bik*, *bax*, *apaf1*; n = 4–6). **G**–**H** Intestinal caspase3, caspase9 activity of zebrafish (n = 4–6). Data are presented as mean ± SEM. Student’s *t*-test was used for statistical analysis, with *p < 0.05, **p < 0.01, and ***p < 0.001

Acidic mucin secreted by goblet cells is stained blue by AB-PAS stain. Supplementation with *A. muciniphila* significantly reversed the HFD-induced reduction in goblet cells (Fig. 2A). HFD induced intestinal chronic inflammation. Our study showed that compared with HFD group, *A. muciniphila* supplementation had no effect on *TNFα* (Fig. 2B), but significantly downregulated *IL-1β* expression (*p* < 0.05; Fig. 2C). The anti-inflammatory cytokine *IL-10* showed an upward trend in expression (*p* = 0.07; Fig. 2D), whereas *TGFβ* expression was significantly increased (*p* < 0.05; Fig. 2E). Consuming high levels of fat induces endoplasmic reticulum (ER) stress of intestines, which further activates apoptosis in fish (Ling et al. 2019). As shown in Fig. 2F, oral administration of *A. muciniphila* significantly down-regulated pro-apoptotic genes *bid* and *bax*, up-regulated anti-apoptotic gene *bcl-2*, and increased the *bcl-2*/*bax* ratio. (*p* < 0.05; Fig. 2F). The activities of caspase3 and caspase9 further confirmed that it alleviated intestinal cell apoptosis in HFD-fed zebrafish (*p* < 0.001, *p* < 0.05; Fig. 2G, H).

**Fig. 3:**
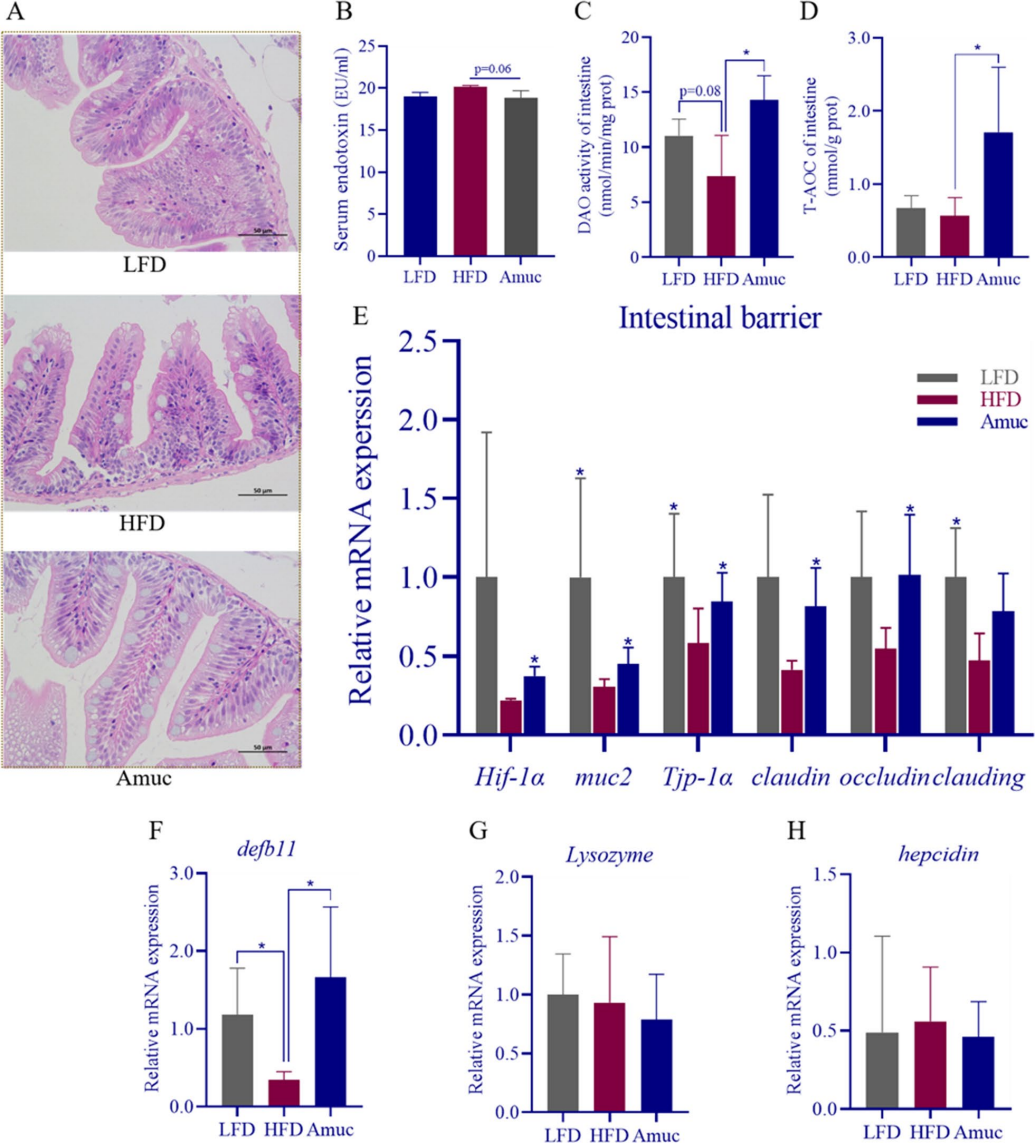
*Akkermansia muciniphila* ameliorates the intestinal barrier function disorder induced by HFD and alleviates endotoxemia. **A** Representative images of H&E stained intestinal histology. Scale bar = 50 μm. **B** serum endotoxin levels. **C** Intestinal DAO activity. **D** Intestinal T-AOC levels (n = 4–6). **E** Expression of intestinal barrier related genes (n = 3–6). **F**–**H** Expression of intestinal antimicrobial peptides (n = 3–6). Data are presented as mean ± SEM. Student’s *t*-test was used for statistical analysis, with *p < 0.05

HFD leads to a compromised intestinal barrier and increased serum endotoxin levels. Firstly, *A. muciniphila* could ameliorate intestinal morphological damage (Fig. 3A). Additionally, oral administration of the bacterium showed a distinct tendency to reduce serum endotoxin level (*p* = 0.06, Fig. 3B). Moreover, *A. muciniphila* has the ability to greatly increase DAO enzyme activity and improve the antioxidant capacity in the intestine (*p* < 0.05, Fig. 3C, D). *q*RT-PCR showed that *A. muciniphila* upregulated intestinal barrier-related genes, including *Hif-1α*, *muc2*, *Tjp-1α*, *claudin1*, *occludin* (*p* < 0.05, Fig. 3E). Intestinal antimicrobial peptides play an important role in intestinal barrier function. The expression of intestinal *defb11* was significantly down-regulated by HFD, and the addition of *A. muciniphila* could significantly reverse it (*p* < 0.05, Fig. 3F). But there was no discernible difference in the expression of *lysozyme* and *hepcidin* in both the HFD and Amuc groups (Fig. 3G, H).

### *Akkermansia muciniphila* alleviates metabolic disorders partially depending on gut microbiota

**Fig. 4:**
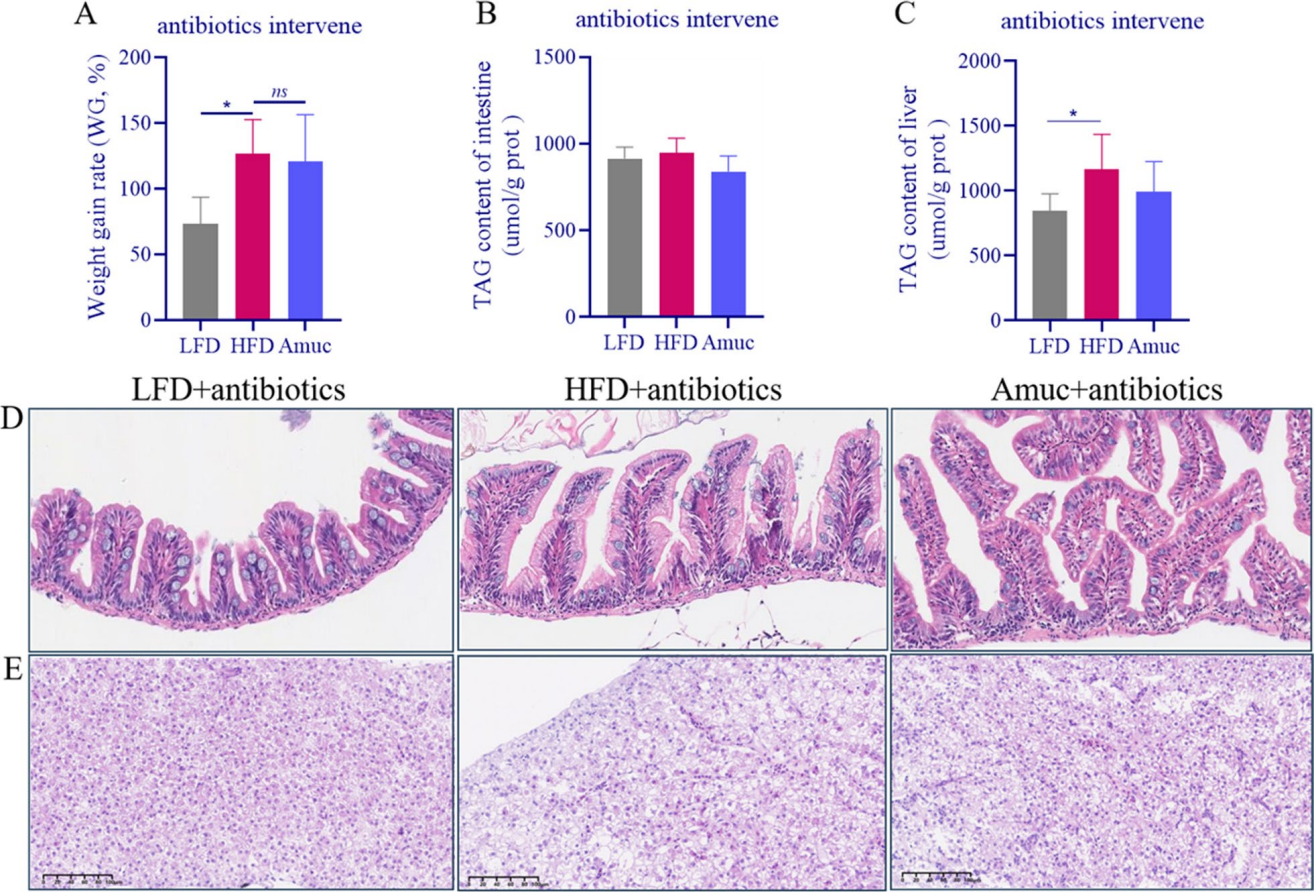
*Akkermansia muciniphila* mitigates liver lipid deposition through partial reliance on gut microbiota. **A** weight gain rate, **B** Liver TAG (n = 3–6), **C** intestine TAG (n = 3–6) content of zebrafish after 4 weeks of feeding in *Experiment 2*. Representative images of H&E stained intestinal (**D**) and liver (**E**) histology (scale bar = 50 μm). Data are presented as mean ± SEM. Student’s *t*-test was used for statistical analysis, with *p < 0.05

To determine whether gut microbiota supports the probiotic effect of *A. muciniphila*-induced metabolic phenotype, we performed a 4-week broad-spectrum antibiotics cocktail treatment on zebrafish. The impact of *A. muciniphila* in lowering HFD-induced weight gain was eliminated following a 4-week intervention (Fig. 4A). Specifically, its effect in alleviating and excessive fat deposition of liver and intestine was also eliminated (Fig. 4B, C). And we also found that *A. muciniphila* cannot effectively reverse the intestinal damage caused by HFD after co-administration of antibiotics (Fig. 4D). Moreover, the liver H&E section showed no change in the alleviation of steatosis after antibiotic intervention (Fig. 4E). This indicated that the metabolic regulation of *A. muciniphila* is affected after depletion of the gut microbiota.

### *Akkermansia muciniphila* application regulated HFD-induced gut microbiota community dysbiosis, elevated the tryptophan metabolizing-related genus

**Fig. 5:**
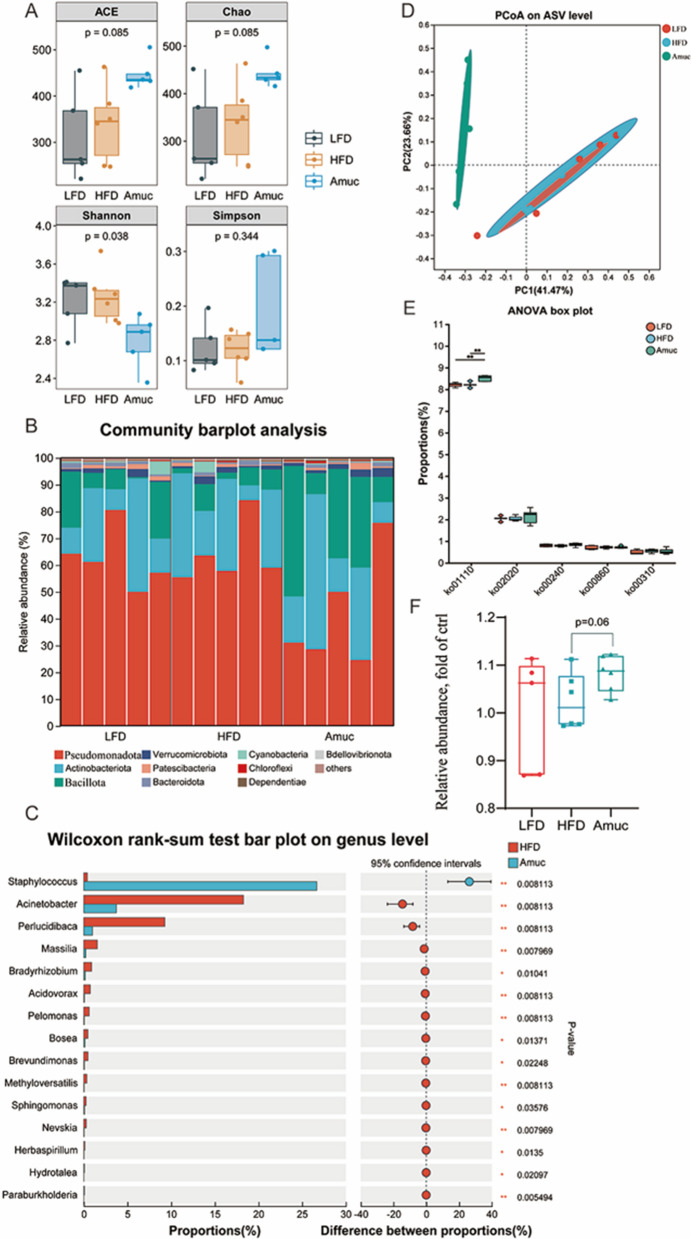
*Akkermansia muciniphila* administration modulate the diversity and structure of gut microbiota. **A** Intestinal microbiota α-diversity analysis (n = 5–6); **B** Stacked Column Chart shows the differences of the composition of different groups of intestinal microbial community at phylum level. **C** Significant differential bacterial genera of gut microbiota was filtered caused by *A. muciniphila* intervention. The *p*-value of significance was based on Wilcoxon rank-sum test comparison between HFD group and Amuc group. **D** Intestinal microbiota β-diversity-Principal coordinate analysis score plots of ASV level in three different dietary groups. **E** The functional differences of bacteria in different groups were analyzed by the KEGG metabolic pathway displayed with the ko number. **F** Differences in the relative abundance of tryptophan metabolizing bacteria among different groups (Student’s *t*-tests)

*Akkermansia muciniphila* colonizes the intestinal mucus layer, digesting mucus to interact with symbiotic microbiota via trophic relationships. It enhances microbial gene richness and ecosystem abundance, and its gut crosstalk may improve obesity-related metabolism (Ling et al. 2019). Intestinal community composition was analyzed via 16S *r*RNA high-throughput sequencing, yielding 1,430,214 optimized sequences clustered into 2655 OTUs. Ace and Chao index showed that microbial richness had an obvious increase, while Shannon index was significantly reduced in the Amuc group (Fig. 5A, Supplementary Table 4). The above results suggested that the administration of *A. muciniphila* may exert a strong regulating effect on the intestinal microbial community. We next analyzed the composition of intestinal microbial community in different groups. At the phylum level, the types of core microbiota were identical in different dietary groups, including *Pseudomonadota*, *Actinobacteriota*, *Bacillota*, *Verrucomicrobiota*, *Bacteroidota* (Fig. 5B, Supplementary Table 5). In contrast to the HFD group, the Amuc group exhibited a significant reduction in the relative abundance of Pseudomonadota (41.1% vs. 58.6%). Conversely, the relative abundance of Bacillota was markedly higher in the Amuc group (29%) than in the HFD group (9.72%). There were no obvious changes in *Bacteroidota* among the three dietary groups. Analysis at the genus level revealed that *A. muciniphila* increased the relative abundance of *Staphylococcus*, *Vibrionacea*e, *unclassified_f_Vibrionaceae*, while decreasing the relative abundance of *Acinetobacter*, *Perlucidica*, *Massilia*, *Acidovoorax* and *Bradyrizobium* (Fig. 5C, Supplementary Table 6). And Principal Coordinate Analysis (PCoA) showed that the intestine microbiota structure of LFD and HFD had relatively high similarity, while there is obvious shifts from the Amuc group (Fig. 5D). Predictive analysis using PICRUSt2 combined with the KEGG database revealed that supplementation of *A. muciniphila* enhanced the function of gut microbiota related to Biosynthesis of Secondary Metabolites (ko01110, *p* < 0.01) and acetate synthesis (ko02020) (Fig. 5E). Further predictive analysis indicated that the supplementation of *A. muciniphila* may increase the abundance of bacterial genera associated with tryptophan metabolism (Fig. 5F).

### *Akkermansia muciniphila* altered the gut metabolite profile and pathways

**Fig. 6:**
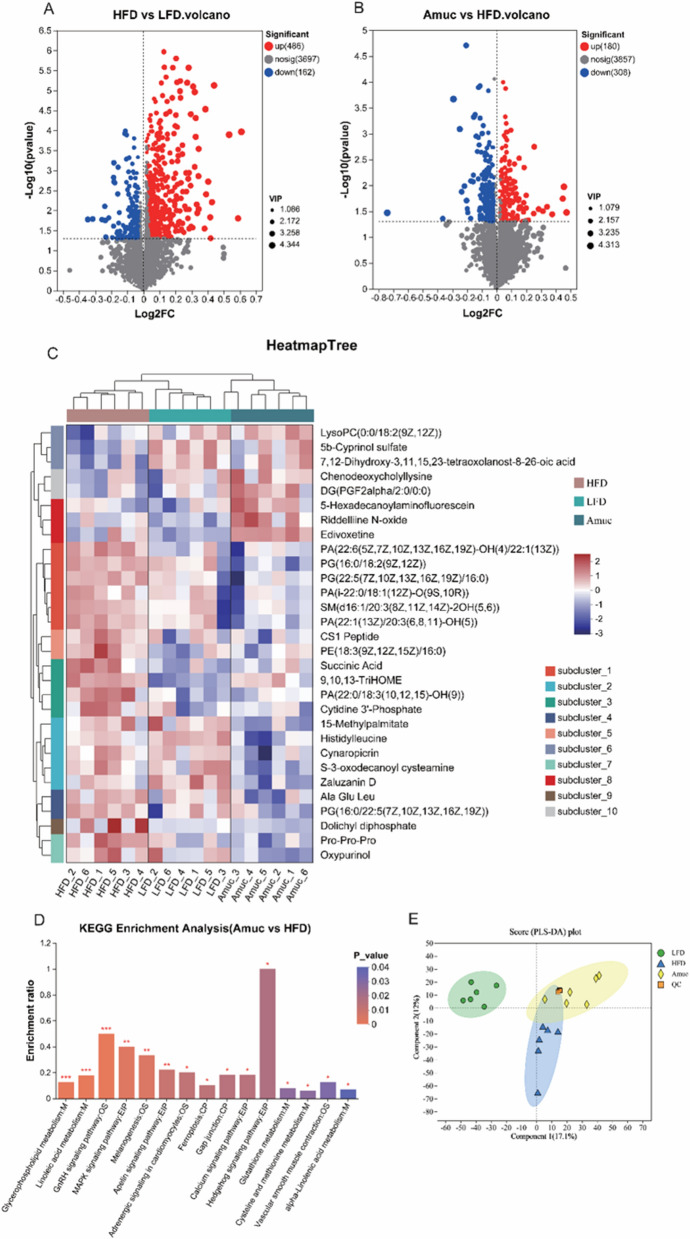
Comparison of metabolite profiles among three different dietary groups (n = 6). **A**, **B** Volcano maps are based on differential metabolite screening compared to HFD vs. LFD and Amuc vs. HFD, the red dots indicate up-regulated metabolites (FC (HFD/LFD, Amuc/HFD) > 1), the blue dots indicate down-regulated metabolites (FC (HFD/LFD, Amuc/HFD) < 1), and the gray dots indicate undifferentiated metabolites with *p* < 0.05. **C** Orthogonal projection score plot of potential structure discriminant analysis of intestinal metabolites (PLS-DA) in different group, quality control (QC). **D** KEGG enrichment analysis (Amuc vs. HFD). **E** Heatmap and clustering tree of fold change of the top 30 differential metabolites in three dietary groups

The gut microbiota affects the metabolites. Therefore, the changes of zebrafish gut metabolites after *A. muciniphila* intervention were investigated by untargeted transcriptomics. We identified a total of 4345 metabolites, which were parameterized (*p* < 0.05, VIP 1.0 and fold change (FC) > 1,) to construct a differential metabolite dataset. The volcano plot showed that HFD had elevated 486 and reduced 162 metabolites when compared to LFD, while the addition of *A. muciniphila* upregulated 180 and downregulated 308 metabolites when compared to the HFD group (Fig. 6A, B). This indicated that *A. muciniphila* administration could significantly alter the gut metabolic profile. Hierarchical cluster analysis (HCA) was performed for the top 30 differential metabolites (Fig. 6C). HCA presented two main clusters, LFD and HFD samples, Amuc and LFD samples clustered separately in a sub-cluster. This indicated the differences in accumulated metabolites as a result of the addition of *A. muciniphila*. The key metabolic pathways caused by different diets were further enriched through KEGG enrichment pathway analysis. As depicted in Fig. 6D, the top 15 metabolic pathways with the highest enrichment were selected from the KEGG database in Amuc group versus HFD group. Among the enriched KEGG pathways, Lipid metabolism (Primary bile acid biosynthesis, Steroid hormone biosynthesis, Linolenic acid metabolism, alpha-Linolenic acid metabolism, Glycerophospholipid metabolism), Endocrine system (Insulin/GnRH signaling pathway), Signal transduction (Apelin/Hedgehog/MAPK signaling pathway), and Gap junction, Ferroptosis pathway were significantly enriched (*p* < 0.05). Furthermore, in the Amuc group, the overall expression of the pathways of Primary bile acid biosynthesis and Insulin signaling pathway tended to be upregulated, while the overall expression of the pathways related to lipid synthesis, such as Linolenic Acid/alpha-Linolenic acid/Glycerophospholipid metabolism, tended to be downregulated. Further two-component PLS-DA models comprehensively showed the metabolome profiles evidently discriminated among the three dietary groups. More specifically, the first two components account for 29.1% of the total variation, with the first component explaining 17.1% and the second 12%, respectively (Fig. 6E).

### ***Akkermansia muciniphila*** shifted tryptophan regulation, activating the AhR

**Fig. 7:**
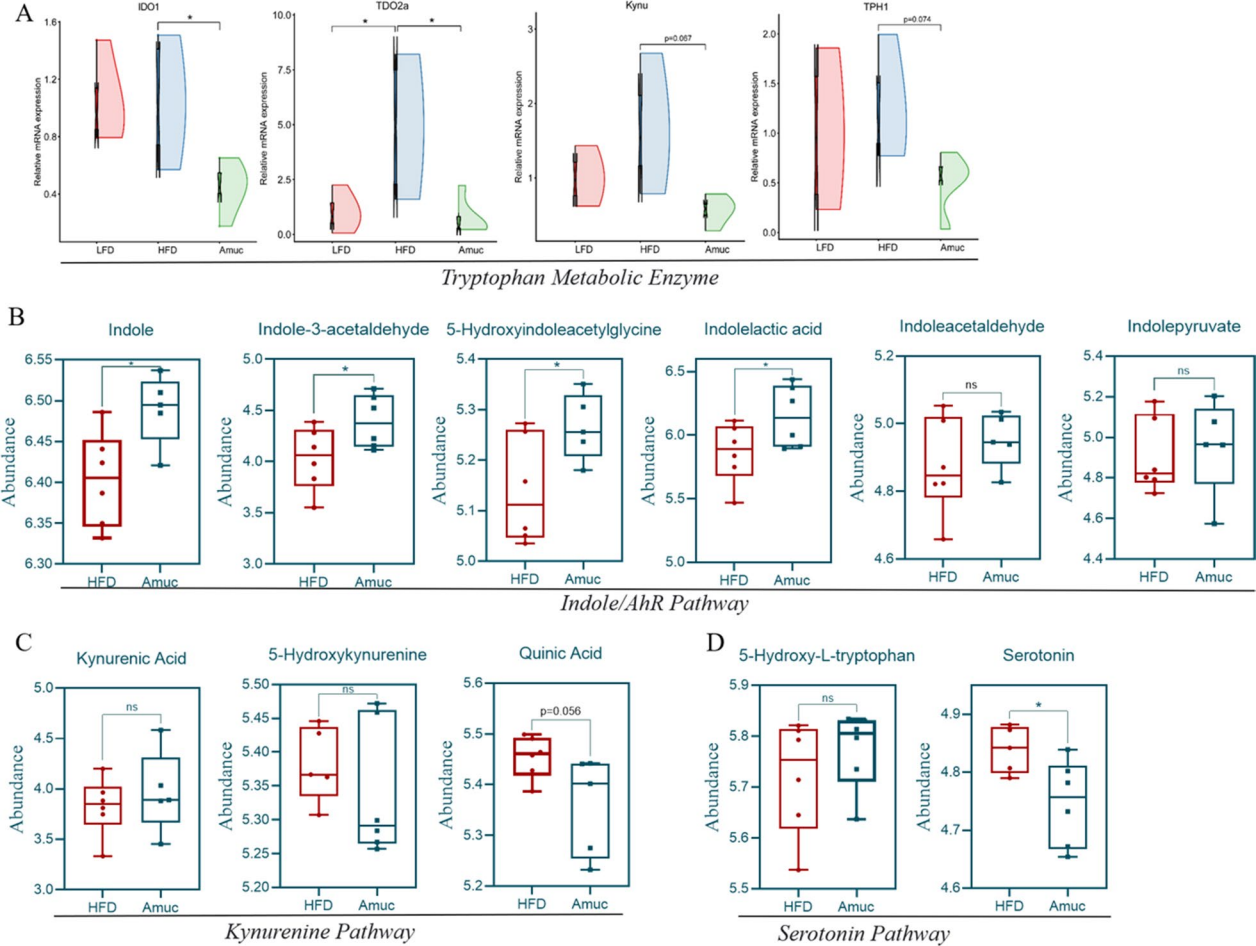
Effects of *A. muciniphila* on tryptophan metabolism enzymes and the abundance of tryptophan-related metabolites in the intestine. **A** The effect of *A. muciniphila* on the indoleamine 2,3-Dioxygenase1(IDO1), tryptophan 2,3-dioxygenase (TDO2a), kynureninase (Kynu) tryptophan hydroxylase 1 (TPH1) gene expression levels in the intestine (n = 4–6). **B**–**D** The effect of *A. muciniphila* on the metabolites of the three major metabolic pathways: *Indole*, *Kynurenine*, *Serotonin pathway* of tryptophan in intestine (n = 4–6). Data are presented as mean ± SEM. Student’s *t*-test was used for statistical analysis, with *p < 0.05

*Akkermansia muciniphila* modulates the community structure of the gut microbiota. It has been demonstrated that three gut microbiota metabolites, including secondary bile acids metabolism, SCFAs metabolism, and tryptophan are crucial to the host's physiological metabolism (Nicolas and Chang 2019). Given that HFD can induce drastic changes in tryptophan metabolism, we conducted a study to investigate whether the administration of *A. muciniphila* has an impact on the pathways associated with the tryptophan-related differential metabolites (Fig. 7). Three main metabolic processes catabolized tryptophan when it enters the gastrointestinal tract: the host kynurenine and serotonin pathway, and the gut microbial system which directly converts tryptophan into its distinct metabolites, indole and IDs (Agus et al. 2018). First, we found that the Amuc group exhibited a marked reduction in the expression of key enzymes in the KP and 5-HT pathways compared to the HFD group, including IDO1 (*p* < 0.05), TDO2a (*p* < 0.05), Kynu (*p* = 0.067), and TPH1 (*p* = 0.074) (Fig. 7A). Tryptophan metabolites were therefore examined both before and after the administration of *A. muciniphila*. We found the metabolites of the AhR pathway, which represents the microbial metabolism of tryptophan, have increased, including indole (*p* < 0.05), indole-3-acetaldehyde (IAAId) (*p* < 0.05), 5-hydroxylindoleacetylglycin (*p* < 0.05), ILA (*p* < 0.05), while indoleacetaldehyde and indolepyruvate had no notable alteration (Fig. 7B). The metabolites in KP such as kynurenic acid and 5-hydroxykynurenine showed no discernible differences, while quinic acid (*p* = 0.056) exhibits a considerable downward trend (Fig. 7C). Serotonin levels significantly decreased although the 5-hydroxy-L-tryptophan in serotonin pathway remained unchanged (Fig. 7D). In summary, the intake of *A. muciniphila* inhibited several key enzymes in the KP and 5-HT pathway, consequently driving the tryptophan metabolic pathway towards a microbiota-dependent direction.

**Fig. 8:**
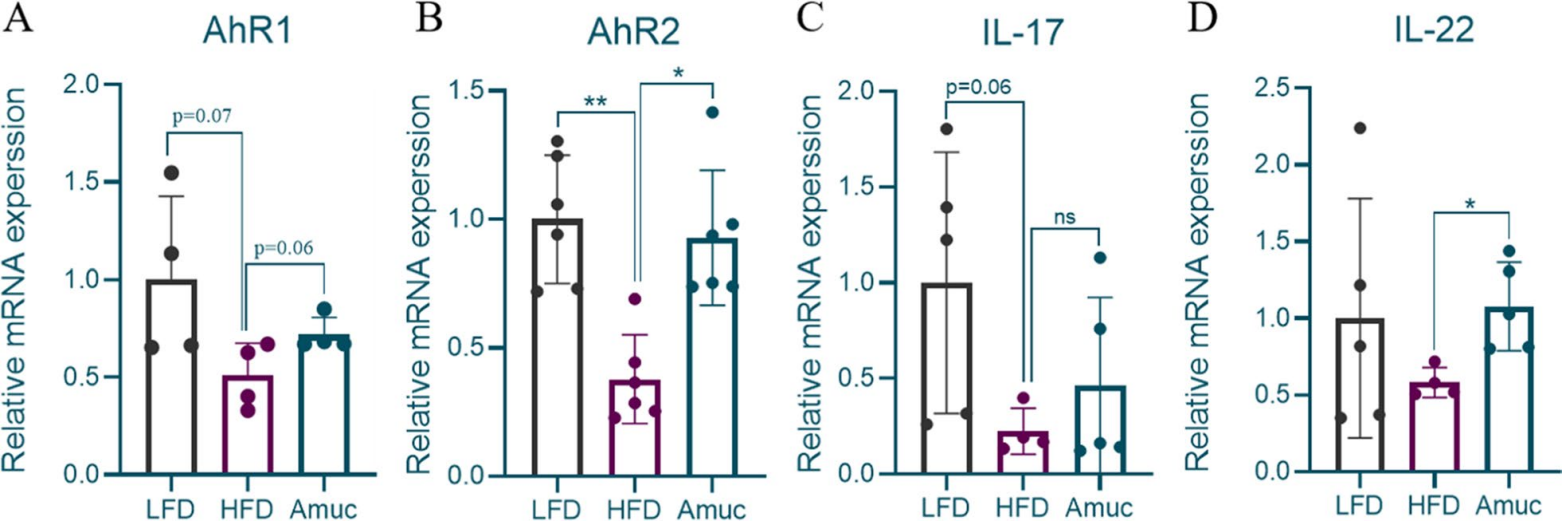
*Akkermansia muciniphila* affects AhR and downstream target genes. The relative expression of AhR1 (**A**), AhR2 (**B**), *IL-17* (**D**), *IL-22* (**E**) at the mRNA level (n = 4–6). **C** Representative western blotting showed the expression of AhR in protein levels among the LFD, HFD, and Amuc groups (n = 3). Data are presented as mean ± SEM. Student’s *t*-test was used for statistical analysis, with *p < 0.05, **p < 0.01

Metabolomics studies confirmed that mucin phage significantly altered tryptophan metabolites. These metabolites, in turn, activate AhR, which plays a role in maintaining intestinal homeostasis and regulating metabolism (Yano et al. 2015). To determine its effect on intestinal AhR signaling, we first conducted detection using *q*RT-PCR. As expected, *A. muciniphila* showed an upward trend in AhR1 expression (*p* = 0.06; Fig. 8A), while it successfully reversed the HFD-induced decrease in *AhR2* expression at the mRNA level (*p* < 0.05; Fig. 8B). To clarify AhR's effect on downstream targets, we detected *IL-17* and *IL-22* expression (Fig. 8C, D). HFD tended to reduce *IL-17* level (*p* = 0.06), while *A. muciniphila* addition significantly increased *IL-22* level (*p* < 0.05). *AhR* plausibly contributes to the interaction between metabolism and the proinflammatory state during the onset of obesity and in T2D patients (Carcia-Villatoro et al. 2017). Moreover, *IL-22* maintains intestinal integrity and barrier functions, and is linked to insulin resistance in obesity. Therefore, *A. muciniphila* alleviates HFD-induced intestinal inflammation and barrier by activating *AhR* and downstream target gene *IL-22*.

### Correlation analysis among intestinal microbiota and metabolites

**Fig. 9:**
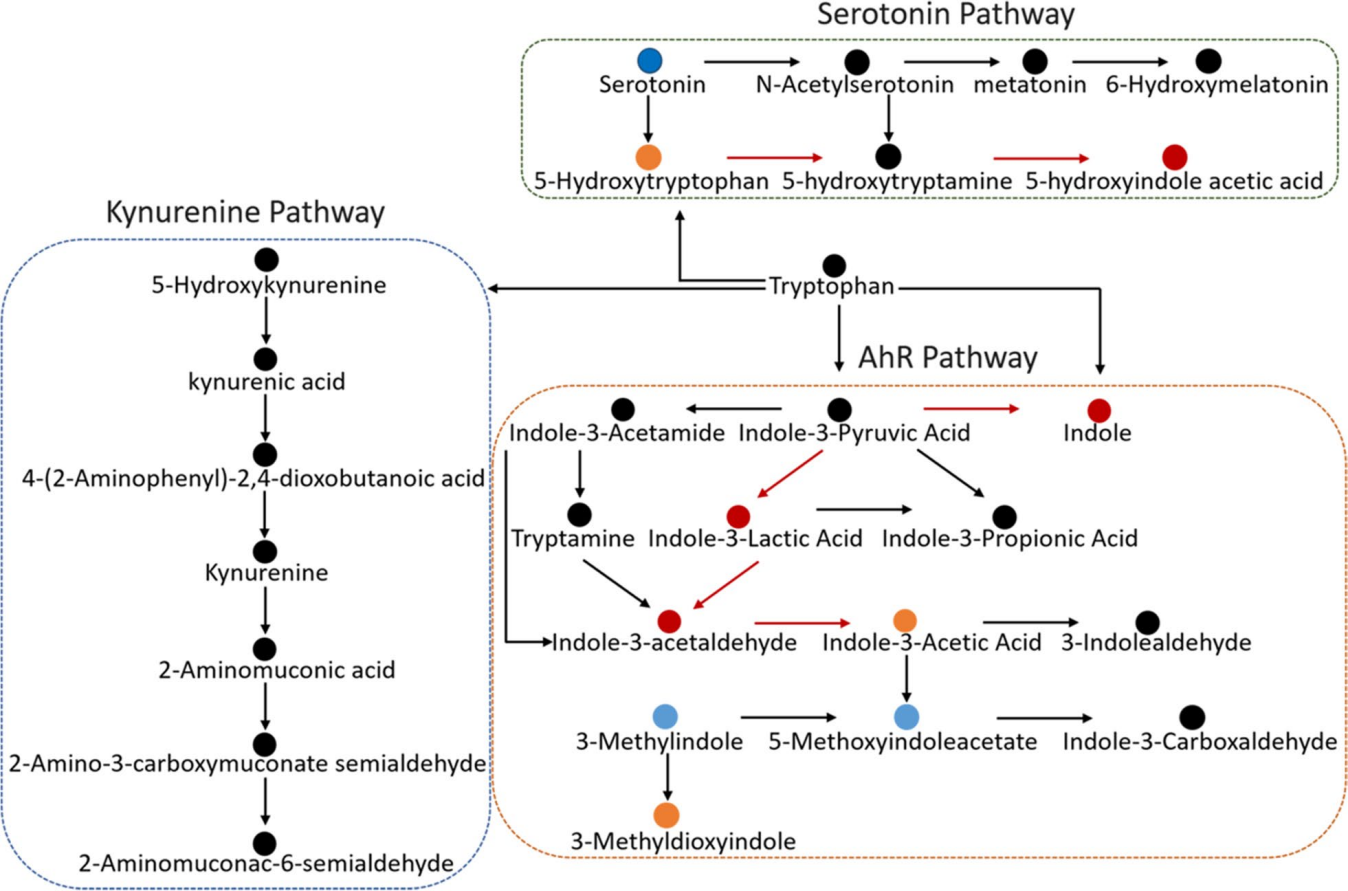
Modulation of tryptophan metabolism by *A. muciniphila* intervention in HFD-fed zebrafish. Schematic representation of three major tryptophan pathways (kynurenine, serotonin, and indole derivatives) in the liver. Metabolite abundance changes between the Amuc and HFD groups are indicated by circle colors: Dark-blue denotes a significant decrease, while light-blue indicates a downward trend. Red represents a significant increase, and yellow implies an upward trend. ‌Red arrows‌ highlight pathways with altered metabolic flux

Subsequently, Fig. 9 provided a clear representation of the outcomes derived from the pathway analysis. We discovered that the treatment with *A. muciniphila* enhanced the gut microbiota's capacity to metabolize tryptophan. This enhancement potentially led to the generation of higher levels of 5-hydroxyindole acetic acid (5-HIAA), ILA, IAAId and indole-3-acetic acid (IAA). Consequently, the *A. muciniphila* intervention potentially augmented the metabolism of tryptophan along the 5-HT pathway and the AhR pathway. In line with the latest and most comprehensive literature accounts, the tryptophan metabolites elevated through these two metabolic pathways are intrinsically dependent on the gut microbiota (Zhang et al. 2024; Du et al. 2024). Therefore, *A. muciniphila*'s activation of AhR and its target gene depends on its regulation of gut microbiota-mediated tryptophan metabolism.

**Fig. 10:**
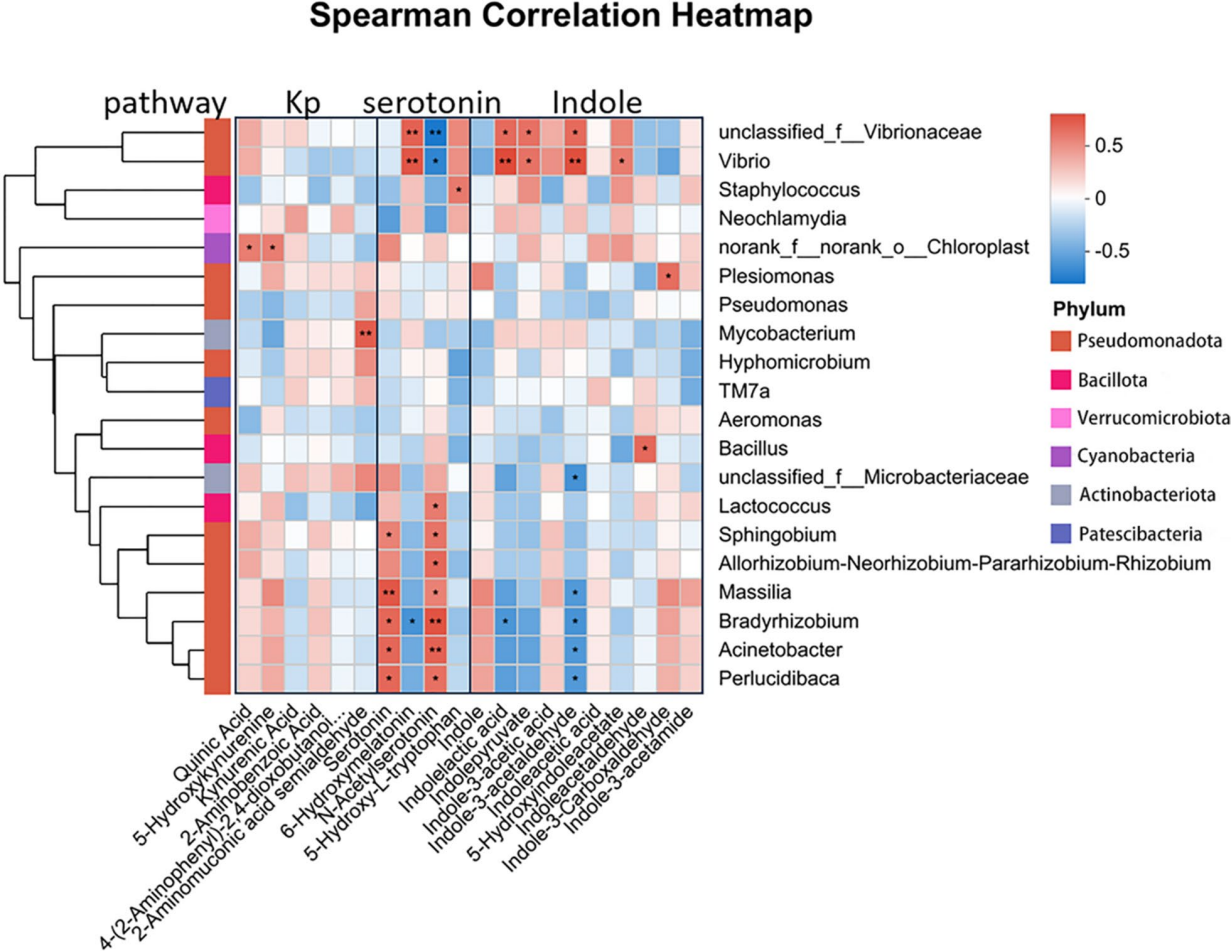
Spearman correlation network between tryptophan-derived metabolites and gut microbiota genera in HFD-induced zebrafish. Comparisons of the relative abundance of metabolites and microbial genus were used to compute Spearman's rank correlation coefficients and the associated *p*-values. The false discovery rate (FDR) was used to adjust for multiple comparisons, with a threshold of 0.05

For a more profound exploration of the microorganisms involved in the regulatory effect of *A. muciniphila* on tryptophan metabolism, Spearman analysis was employed to establish the connection between the altered bacteria and tryptophan-related differential metabolites. As depicted in Fig. 10,

treatment with *A. muciniphila* up-regulated indole, indolepyruvate, IAAId, and 5-HIAA, which positively correlated with *Plesiomonas*, *Vibrio*, *unclassified_f_Vibrionaceae*, and *Staphylococcus*. Specifically, relative abundances of these bacterial taxa were elevated in the Amuc group compared to HFD group (*p* = 0.1; *p* < 0.05; *p* < 0.05; *p* < 0.01). Nevertheless, quinic acid and kynurenic acid, which are products of the host's tryptophan metabolic pathway, either had no association or a negative correlation with these bacteria. In contrast, they showed a positive correlation with *chloroplasts*. Furthermore, a negative correlation was detected between ILA and both *Bradyrhizobium* and *Massilia*. Remarkably, the relative abundances of these two bacterial taxa were substantially reduced in the Amuc group (*p* < 0.01; *p* < 0.05). In contrast, serotonin levels were down-regulated in the Amuc group. This neurotransmitter displayed a significant positive correlation with *Massilia*, *Bradyrhizobium*, *Acinetobacter*, *Perlucidibaca*, and *Sphingobium*. Significantly, the Amuc group demonstrated considerably reduced relative abundances of these bacterial taxa in comparison to the HFD group (*p* < 0.05). *Bacillus* and *Lactococcus* exhibited a positive correlation with indoleacetaldehyde and N-acetylserotonin, respectively. The abundances of these two bacterial genera were decreased in the Amuc group. In summary, *A. muciniphila* may affect tryptophan metabolism by increasing the abundance of *Plesiomonas*, *Vibrio*, *unclassified_f_Vibrionaceae*, and *Staphylococcus* while decreasing the abundance of *Bradyrhizobium*, *Massilia*.

## Discussion

With the improvement of living standards and changes in lifestyle, the increasing intake of carbohydrates and lipids has led to a rising incidence of chronic metabolic diseases. Currently, the global prevalence of MASLD in the adult population has reached approximately 32%, making it the primary chronic liver disease at present (Teng et al. 2023). Over the past two decades, the incidence of MASLD has increased seven-fold, becoming a primary and prevalent driver of global liver-related deaths (Allen et al. 2018).

MASLD is primarily driven by overnutrition and insulin resistance, leading to hepatic lipid droplet accumulation, ectopic fat, and metabolic abnormalities (Paik et al. 2020). Current research indicates that in addition to hepatic steatosis, MASLD is accompanied by a series of systemic metabolic disorders, including endoplasmic reticulum stress, inflammatory response, bacterial translocation caused by gut microbiota dysbiosis, and the consequent endotoxemia (Zhao et al. 2020; Zimmermann et al. 2019; Utzeri and Usai 2017). Thus, the pathogenesis of MASLD has gradually moved beyond the "two-hit hypothesis" proposed by British scholar Day in 1998 to the current “multiple-hit hypothesis” (Day and James 1998; Loomba et al. 2021). The disease in MASLD patients progresses slowly, making it difficult to conduct continuous clinical dynamic observation and sampling. Therefore, research on the pathogenesis of MASLD, drug screening, and target evaluation all rely on appropriate experimental animal models. Currently, the pathogenic factors of diet-induced models in rodents are similar to those of human MASLD. Rats and mice have been widely used in MASLD efficacy evaluation experiments, but it takes a long time to establish the models (Van et al. 2017; Zhao et al. 2022).

In recent years, zebrafish, as a new type of model organism, has been widely used in research on human diseases (Liu 2022; Chang et al. 2023). Zebrafish are small in size, easy to breed, and have a high reproductive rate, which can significantly shorten the test cycle. Zebrafish embryos develop rapidly and are transparent throughout the development process, which is conducive to in vivo research on development and nutritional metabolism (Siriyappagouder et al. 2020). Notably, zebrafish share 87% genomic homology with humans, with their genomes fully sequenced. At present, researchers have established zebrafish fatty liver models through gene knockout and diet-induced methods (Zhao et al. 2022; Asaoka et al. 2013; Fan et al. 2020). In our prior study, a zebrafish model of nutritional hepatic steatosis was successfully established by feeding 1-month-old zebrafish a 16% high-fat diet for 4 consecutive weeks (Li et al. 2024; Zhang et al. 2022; Zhang et al. 2023a, b). Using this zebrafish model, we are systematically exploring *A. muciniphila*'s therapeutic potential in ameliorating hepatic steatosis and enhancing gut barrier health, aiming to elucidate novel protective mechanisms via integrated molecular biology and multi-omics approaches.

In this work, therapeutic supplementation with *A. muciniphila* at 10^8^ cfu/g significantly attenuated pathological TAG accumulation, and it reduced the HFD-induced body weight gain and mortality. To explore the molecular mechanism by which *A. muciniphila* regulates hepatic lipid metabolism, we found that it significantly reduced the expression of key enzymes in lipid synthesis, such as *PPARγ*, *C/EBPα*, *FAS*, *ACC1*, and *DGAT2*. However, it had no effect on β-oxidation. Its regulatory effect on lipid metabolism in zebrafish closely resembles that in HFD-induced mice (Everard et al. 2013; Plovier et al. 2017). In addition, we found that the treatment with *A. muciniphila* had no effect on muscle fat. Zhao et al. (2017) showed that it could reduce muscle fat by reducing the gene expression related to fatty acid synthesis (Zhao et al. 2017). This difference might be due to the use of a different model. Meanwhile, the administration of *A. muciniphila* can significantly alter the gut metabolic profile. Consistent with hepatic metabolic remodeling, ‌*A. muciniphila‌* intervention induced significant enrichment of intestinal lipid catabolism pathways.

The gut-liver axis's role in MASLD pathogenesis is elaborated, emphasizing that the intestinal barrier-an integral part of this axis-protects the liver by blocking intestinal noxious substance translocation to it (Albillos et al. 2020). The intestinal barrier comprises four interconnected layers: (a) mechanical barrier (epithelial tight junctions), (b) chemical barrier (mucus and antimicrobial peptides), (c) immune barrier (sIgA and immune cells), and (d) biological barrier (gut microbiota) (Scaldaferri et al. 2012). Oral *A. muciniphila* degrades intestinal mucus, compensatorily normalizes inner mucus layer thickness, and enhances intestinal barrier function (Grander et al. 2018). However, it has also been reported that *A. muciniphila* can exacerbate mucosal barrier damage during intestinal infection or inflammation (Marcella et al. 2021). In our study, *A. muciniphila* effectively reversed HFD-induced damage to intestinal mechanical and chemical barriers, significantly upregulating tight junction proteins *Tjp-1α*, *claudin1*, and *occludin*, as well as that of *muc2* and *defb11*. This is consistent with morphological observations. Overall, this indicates that in the context of chronic inflammation induced by HFD, *A. muciniphila* improve the intestinal barrier and simultaneously reduce the inflammatory response.

Increasing evidence suggests that gut dysbiosis increases the risk of obesity and MASLD. Moreover, Gram-negative (G-) bacteria are prevalent in MASLD, especially *Pseudomonadota* (Filipovic et al. 2021; Vasques-Monteiro et al. 2021). Furthermore, given that *Pseudomonadota* are recognized as one of the premier sources of lipopolysaccharide (LPS), the increase in their proportion further leads to the occurrence of endotoxemia in mice fed HFD (Miura et al. 2017). As endotoxin travels through the bloodstream to reach the liver, it influences the liver's inflammatory and metabolic dysfunction. LPS stimulates pro-inflammatory cytokines, thereby perpetuating low-grade inflammation in metabolic disorders (Mohammad and Thiemermann 2021). Furthermore, inflammation-induced intestinal leakage of gut microbial endotoxins serves as a key driver of hepatic steatosis. This process suppresses hepatic fatty acid β-oxidation, impairing metabolism, promoting hepatic accumulation, and exacerbating lipid deposition (Henao-Mejia et al. 2023). In line with prior research, our study found that HFD led to a marked enrichment of *Pseudomonadota* (58.5%) in zebrafish, whereas *A. muciniphila* supplementation reversed this trend, achieving a 17% reduction in its relative abundance. At the genus level in particular, a significant decrease was mainly manifested in *Acinetobacter* and *Perlucidibaca*. Correspondingly, HFD significantly increased the serum endotoxin level, which was significantly improved after *A. muciniphila* treatment. The findings indicated that *A. muciniphila* mitigates hepatic steatosis and inflammation by enhancing the integrity of the intestinal barrier, particularly by restoring gut microbiota dysbiosis. Moreover, through the intervention of antibiotics, we further demonstrated that this probiotic effect is mediated by the gut microbiota. Of course, this inference needs to be further confirmed by subsequent studies using germ-free zebrafish and zebrafish larval microbiota transplantation models. Furthermore, based on 16S *r*RNA sequencing results-showing *A. muciniphila*’s regulatory effect on gut microbiota-and the loss of its effect post-antibiotic intervention, *A. muciniphila* may also regulate zebrafish lipid metabolism by mediating the microbiota. The latent mechanism involving the joint participation of multiple bacteria requires further in-depth study.

SCFAs play an important role in improving metabolic disorders (Nicolas and Chang 2019). Metabolomic analysis in this study showed that the detected level of SCFAs in mixed intestinal samples of zebrafish was relatively low; therefore, the research focus was shifted to another group of host metabolism-related substances: tryptophan-gut microbiota metabolites. Studies show that the gut microbiota can metabolize tryptophan in three different manners, ‌KP‌: ~ 90% is catabolized by intestinal epithelial/immune cells; 5-HT pathway‌: ~ 3% converted to 5-hydroxytryptamine in enterochromaffin cells; Indole pathway‌: 4%-6% directly metabolized to indole derivatives (Gao et al. 2020). Dynamic equilibrium among these pathways maintains tryptophan metabolic homeostasis. ‌Notably, HFD significantly reduces microbial production of tryptophan-derived metabolites‌, including tryptamine and indole-3-acetate (I3A), thereby aggravating‌ HFD-associated chronic inflammatory responses (Krishnan et al. 2018). ‌‌Furthermore, fecal analyses revealed reduced IDs and elevated kynurenine levels in patients with obesity and metabolic syndrome. Consistently, HFD-fed mice exhibited persistent gut microbiota dysbiosis, followed by a reduction in tryptophan-derived microbial metabolites (Aron-Wisnewsky et al. 2021). This indicates that gut microbiota-dependent metabolites (IDs) contribute to metabolic disease pathogenesis, exhibiting an inverse association (Bock 2020). Our gut microbiota sequencing results show that *A. muciniphila* treatment effectively reverses HFD-induced dysbiosis. Thus, we speculate that it may improve HFD-induced tryptophan-indole pathway metabolism in zebrafish. Metabolomic analysis showed that *A. muciniphila* could indeed reverse the decrease in IDs induced by HFD, such as indole, indole-3-acetaldehyde, 5-hydroxyindoleacetylglycine, 5-hydroxyindole-3-acetic acid and ILA. Meanwhile, the levels of quinic acid and serotonin decreased. This indicated that the *A. muciniphila* intervention impacts the balance and proportion of the three major tryptophan metabolic pathways, enhancing the microbiota-dependent indole pathway while inhibiting the KP and partly 5-HT pathways.

IDs act as a ligands, with binding activating AhR signaling. As expected, *q*RT-PCR showed significant upregulation of two AhR transcripts. As a nuclear transcription factor, it regulates energy metabolism, inflammatory response, lipid and intestinal homeostasis via controlling the transcriptional processes of downstream genes. It can regulate the expression of key enzymes involved in hepatic gluconeogenesis (such as PEPCK), activate thermogenesis in brown adipose tissue, and improve obesity-related metabolic syndromes (Xu et al. 2015). Activation of AhR also leads to increased expression of the *IL-22*-*STAT3* axis in innate lymphoid cells (ILCs) and T cells, enhances epithelial barrier function and the production of antimicrobial peptides (Qiu et al. 2012). Moreover, AhR activation regulates immune cell balance (e.g., Treg/Th17 ratio) to inhibit inflammation, stimulates goblet cells to secrete mucin, and upregulates claudin4 and occluding (Gu et al. 2024). Meanwhile, it promotes the secretion of *IL-22* to maintain barrier integrity, reduce intestinal permeability, and inhibit the intestinal NF-κB inflammatory pathway (Shinde and Tracy 2018). In our study, we found that *A. muciniphila* exhibited significant upregulation of *IL-22* expression, a cytokine essential for maintaining epithelial integrity. Collectively, *A. muciniphila*'s protection against HFD-induced metabolic disruptions is partly due to AhR activation via these tryptophan metabolites, enhancing barrier integrity-related gene differentiation and expression (*muc2*, *Tjp-1α*, *claudin1*, *occludin*). Certainly, future studies should include AhR overexpression and in vivo knockdown experiments to determine the role of *A. muciniphila* in this pathway.

Recent studies indicate that *Burkholderia* species carry out the microbial conversion of tryptophan into 5-HIAA via a non-traditional pathway, and then activates the AhR/TSC2/mTORC1 axis to improve glucose intolerance and obesity (Du et al. 2024). *Bifidobacterium bifidum* and *Lactobacilli* (*Lactobacillus* spp.) encode indole lactate dehydrogenase (aldh), thus having the ability to synthesize ILA (Cui et al. 2023; Wang et al. 2023). ILA activates AhR, promoting tight-junction proteins (*ZO-1*, *occludin*) to enhance intestinal tight junctions, while inducing Treg differentiation and upregulating inhibitory cytokines (*IL-10*, *TGFβ*) to alleviate intestinal inflammation. *Akkermansia muciniphila* mediates the gut microbiota and regulates tryptophan metabolism, thus alleviating the metabolic syndrome induced by HFD. To further investigate which specific microbial species are associated with tryptophan metabolism, Spearman correlation analysis was applied. We found that the upregulated indole derivatives such as indole, indolepyruvate, indoleacetamide (IAAId), ILA and 5-HIAA were positively correlated with *Plesiomonas*, *Vibrio*, *unclassified_f_Vibrionaceae*, and *Staphylococcus*. Meanwhile, the downregulated serotonin level was significantly positively correlated with *Acinetobacter, Massilia*. Consistently‌, Nuidate et al. (2015) demonstrated that *Vibrio cholerae* utilizes the ‌tryptophanase (TrpAse)‌ encoded by the tnaA gene to catalyze the conversion of tryptophan to indole, a key metabolic pathway in bacterial signaling and host interactions (Nuidate et al. 2015). However, bacterial strains associated with ILA‌ and 5-HIAA exhibit discrepancies compared to previous studies. ‌This inconsistency may arise from the distinct composition of the zebrafish gut microbiota‌, which lacks certain dominant taxa (e.g., *Bacteroides*). Certainly, correlation analysis alone has limitations. Further experimental validation-such as in vitro isolation of bacterial strains and spectrometric identification-is required to confirm the specific microbial origin of metabolites, thereby establishing precise correlations between specific intestinal microbes and metabolites.

## Conclusion

This study demonstrates that *A. muciniphila* alleviates HFD-induced metabolic dysfunction, such as disturbances in host lipid homeostasis, intestinal integrity, and inflammatory responses, through gut microbiota-dependent regulation of tryptophan. Specifically, *A. muciniphila* reshapes gut microbiota composition (enriching *Staphylococcus* and *Vibrionaceae* while depleting *Acinetobacter* and *Massilia*), enhances microbiota-dependent tryptophan metabolism (elevating ILA and 5-HIAA). Concurrently, it inhibits the pro-inflammatory kynurenine pathway, shifting tryptophan metabolism toward beneficial microbial-derived metabolites. These metabolic shifts ‌subsequently activate‌ *AhR*, driving the upregulation of *IL-22* and *muc2*, *Tjp-1α*, *claudin1*, and *occludin*, thereby restoring the barrier function, alleviating chronic inflammation and improving hepatic steatosis. These findings establish a novel mechanistic link between *A. muciniphila*-mediated microbiota remodeling, tryptophan metabolic reprogramming, and AhR activation. However, further studies on the mechanism are still needed using germ-free zebrafish models, microbiota transplantation, in vitro microbiota isolation, as well as gene knockout or overexpression approaches.

## Supplementary Information

Supplementary file 1

## Data Availability

The datasets presented in this study can be found in online repositories. The names of the repository/repositories and accession number(s) can be found below: https://www.ncbi.nlm.nih.gov/search/all/?term = PRJNA1094899 And further enquires can be made to the corresponding author.
