# Supplementary material for: Akkermansia muciniphila alleviates metabolic disorders through gut microbiota-mediated tryptophan regulation
Source: AMB Express. 2025 Nov 22;15:181. doi: 10.1186/s13568-025-01986-3 (PMC12748435; doi:10.1186/s13568-025-01986-3)
Supplement: Supplementary file 1 [file 13568_2025_1986_MOESM1_ESM.docx]

Supplementary Figure 1. (n = 3, pool of four zebraﬁsh per sample). Effects of different concentrations of *A. muciniphila* on abnormal accumulation of TAG in liver of zebrafish induced by HFD. Data are presented as mean ± SEM. Student’s t-test was used for statistical analysis, with **p* < 0.05.

**Supplementary Table 1.** Ingredient and nutrient composition of three different types of diet of one month old zebrafish.

| Ingredients (g/kg diet) | LFD | HFD | Amuc |
| --- | --- | --- | --- |
| Casein | 400 | 400 | 400 |
| gelatin | 100 | 100 | 100 |
| dextrin | 280 | 160 | 160 |
| Lard oil | 30 | 80 | 80 |
| Bean oil | 30 | 80 | 80 |
| Lysine | 3.3 | 3.3 | 3.3 |
| VC lecithin | 1 | 1 | 1 |
| Vitamin premix^a^ | 4 | 4 | 4 |
| Mineral premix^b^ | 4 | 4 | 4 |
| Calcium Dihydrogen Phosphate | 20 | 20 | 20 |
| Choline Chloride | 2 | 2 | 2 |
| Sodium Alginate | 20 | 20 | 20 |
| Microcrystalline cellulose | 40 | 40 | 40 |
| Zeolite powder | 65.7 | 85.7 | 85.7 |
| *A. muciniphila* |  |  | 10^^8^ cfu/g |
| Total | 1000 | 1000 | 1000 |
| Crude protein | 421.9 | 421.9 | 421.9 |
| Crude lipid | 60.1 | 160.1 | 160.1 |

Vitamin premix^a^ and Mineral premix^b^ were obtained from Beijing Xinlu United Aquatic Products Co., Ltd. The nutrition provided by the feed meets NRC standards.

**Supplementary Table 2.** Primers sequences about lipid and tryptophan metabolism, inflammatory, barrier function, and apoptosis-related genes for *q*RT-PCR analysis.

| Gene name | Nucleotide sequence of primers (5′-3′) | Size, GenBank accession No. |
| --- | --- | --- |
| *Rps11* | F: ACAGAAATGCCCCTTCACTG  R: GCCTCTTCTCAAAACGGTTG | 146bp, >NM_213377 |
| *PPARγ* | F: CCTGTCCGGGAAGACCAGCG  R: GTGCTCGTGGAGCGGCATGT | 109bp, >NM_131467.1 |
| *C/EBPα* | F: AACGGAGCGAGCTTGACTT  R: AAATCATGCCCATTAGCTGC | 250bp, >NM_131885.2 |
| *ACC1* | F: GCGTGGCCGAACAATGGCAG  R: GCAGGTCCAGCTTCCCTGCG | 137bp, >XM_021476200.1 |
| *FAS* | F: GGAGCAGGCTGCCTCTGTGC  R: TTGCGGCCTGTCCCACTCCT | 128bp, >XM_009306806.3 |
| *SREBP-1α* | F: CAGAGGGTGGGCATGCTGGG  R: ATGTGACGGTGGTGCCGCTG | 150bp, >NM_001030196.1 |
| *PPARα* | F:CTGCGGGACATCTCTCAGTC  R: ACCGTAAACACCTGACGAC | 150bp, >NM_001102567.1 |
| *UCP2* | F: TGCCACCGTGAAGTTTATTG  R: CCTCGATATTTCACCGGACC | 150bp, >NM_131176.1 |
| *TNFα* | F: AAGGAGAGTTGCCTTTACCG  R: ATTGCCCTGGGTCTTATGG | 152bp, >NM_212859.2 |
| *TGFβ* | F: AAAGCAAACCAGCAGAGCAC  R: GCAAAGATAAAGCCAAAATG | 240bp, >NM_182873.1 |
| *IL-10* | F: TCACGTCATGAACGAGATCC  R: CCTCTTGCATTTCACCATATCC | 151bp, >NM_001020785.2 |
| *IL-1β* | F: GGCTGTGTGTTTGGGAATCT  R: TGATAAACCAACCGGGACA | 218bp, >NM_212844.2 |
| *Hif-1a* | F: AGCCGCCACACTTTAGACAT  R: CCTCTGGATCAAAACCCAAG | 76bp, >NM_001308559.1 |
| *Muc2* | F: AATATGCCTTGCGGAACAAC  R: GTGCTGAGGTTGCAGAATGA | 237bp, >NM_001002624.1 |
| *Tjp-1a* | F: CAAAGACCAACAGCACTGCC  R: GTGGTTTAGCGGTGATGGGA | 177bp, >XM_021477863.1 |
| *claudin1* | F: TGTTCATCACTGGAGGGCTT  R: GGAGGATACGAGGGTTTTTC | 209bp, >NM_131770.1 |
| *occludin* | F:TGGAGATGAGCTTGACACAGAT  R: CCTTCCTCTAGCCTGTCGAG | 177bp, >NM_001008618.1 |
| *claudin2* | F: TGATGGGTTTCTTTTTTGGC  R: GAGGGTGTTGTAAGTCTCGC | 188bp, >NM_001004559.2 |
| *defb11* | F: AGGATGCAGCCTCATTCTCTTT  R: TGAAGCCCCAGAGCATATTTAT | 78bp, >NM_001081553.1 |
| *lysozyme* | F: GATTTGAGGGATTCTCCATTGG  R: CCGTAGTCCTTCCCCGTATCA | 103bp, >NM_139180.1 |
| *hepcidin* | F: CACAGCCGTTCCCTTCATAC  R: AGTATCCGCAGCCTTTATTG | 194bp, >NM_001289794.1 |
| *bcl2* | F: GTGGATGATGCACAGGATGT  R: CCTCCAGCTTGGATTGGTTA | 121bp, >NM_001002461.1 |
| *bax* | F: CTGTGTGACCCCAGCCATAAA  R: GATGACAAGGCGACAGGCAA | 216bp, >NM_131562.2 |
| *mcl-1a* | F: AACTCCATCACGCCATACC  R: TCTGCTCAGCCACCCTCT | 270bp, >NM_131599.1 |
| *bid* | F: TGGTGCTCCTTTCCTTTCTT  R: AGGTCGCTGGTGGACTATGT | 147bp, >NM_001079826.1 |
| *bad* | F: CGATGAATGAGGAGGACTTGC  R: CTCCAAAGAAATGCCAACCAG | 235bp, >NM_131579.2 |
| *bik* | F: GGGGACGAAATGGACAATAAA  R: CTGCGAGACCAGTCAGAAAC | 251bp, >NM_001045038.2 |
| *Apaf1* | F: AGTTCTTCTGACCACACGCAA  R: CCTGTTCTGGGAGTTTGTGC | 152bp, >XM_068219731.1 |
| *AhR1* | F:TAGACAGCGATATACAGCAG  R: TCTCTCCAACACCATTCATG | 213bp, >XM_068213883.1 |
| *AhR2* | F: ACGGTGAAGCTCTCCCATA  R: AGTAGGTTTCTCTGGCCAC | 224bp, >NM_131264.1 |
| *IL-17* | F: CGAGAGCCTGTATCCTAC  R: CGTAATCCTGGACCTCAA | 86bp, >NM_001020787.1 |
| *IL-22* | F: CATCGAGGAACAACGGTGTAC  R: CACGAGCACAGCAAAGCAAT | 100bp, >NM_001020792.1 |
| *IDO1* | F: CTCTGCGTGACTTTGTGCTC  R: GGTGGTTCTTGGTTTCCGTT | 322bp, >NM_001083854.1 |
| *TDO2a* | F: CTGTTGCTTGACCAGTTCGC  R: CATCTCCTCCTTCCTCTCCG | 390bp, >NM_001102616.2 |
| *Kyru* | F: GTCCAATCAGCCAATCCTA  R: CTCCAGCTCCTCAAACACA | 265bp, >XM_009302082.4 |
| *TPH1a* | F: CCGCTGCTCATCAAACACT  R: AAACACACGGAAAGCCAAT | 159bp， >NM_178306.3 |

**Supplementary Table 3.** Effect of *A. muciniphila* supplementation on growth performance of zebrafish.

| Parameter | diet | | | |
| --- | --- | --- | --- | --- |
|  | LFD | HFD | Amuc | |
| Initial body weight (IBW, mg) | 45.4±1.34^a^ | 44.8±2.38^a^ | 44.8±2.58^a^ | |
| Final body weight (FBW, mg) | 109.67±6.3^a^ | 115.65±8.2^a^ | 109.56±7.9^a^ | |
| WGR (%) | 153.10±14.55^a^ | 166.89±18.97^a^ | 152.82±18.31^a^ | |
| SGR (%/d) | 3.15±0.14^a^ | 3.38±0.25^a^ | 3.20±0.42^a^ |  |
| Feed conversion Ratio (FCR) | 2.09±0.19^a^ | 1.92±0.20^a^ | 2.11±0.28^a^ | |
| SR (survival rate, %) | 93±2.74^a^ | 86±5.48^b^ | 91±4.18^ab^ | |

The values in the table are given in mean ± SEM. The same letter indicates no significant difference between the two groups, while different letters indicate significant differences between the two groups (*p <* 0.05).

**Supplementary Table 4.** The influence of *A. muciniphila* on the α-diversity indexes of intestinal flora of zebrafish.

| Category | LFD | HFD | Amuc |
| --- | --- | --- | --- |
| ACE | 312.32±96.87^a^ | 338.82±82.32^a^ | 447.80±33.72^b^ |
| Chao | 312.07±96.24^a^ | 338.95±82.87^a^ | 443.51±31.61^b^ |
| Shannon | 3.21±0.28^a^ | 3.25±0.28^a^ | 2.79±0.28^b^ |
| Simpson | 0.12±0.046^a^ | 0.12±0.036^a^ | 0.19±0.09^a^ |
| Coverage | 0.999±0.00 | 0.999±0.00 | 0.999±0.00 |

Different groups of data were analyzed using one-way ANOVA based on Duncan's test to compare their significant differences. And data was represented the means (±SEM), the different letters on each line represent significant differences in different groups.

**Supplementary Table 5.** The predominant gut bacteria at phyla level in zebrafish among three group.

| Phyla | LFD | HFD | Amuc |
| --- | --- | --- | --- |
| *Pseudomonadota* | 59.78±15.88^a^ | 58.55±15.19^a^ | 41.05±24.50^b^ |
| *Actinobacteriota* | 25.79±21.56^a^ | 29.60±14.61^a^ | 28.93±23.67^a^ |
| *Bacillota* | 11.73±9.56^a^ | 9.72±9.04^a^ | 28.99±18.63^b^ |
| *Cyanobacteria* | 1.50±2.23^a^ | 0.96±1.94^a^ | 0.43±0.25^a^ |
| *Bacteroidota* | 0.50±0.19^a^ | 0.52±0.29^a^ | 0.50±0.03^a^ |
| *Verrucomicrobiota* | 0.05±0.04^a^ | 0.01±0.016^a^ | 0.02±0.007^a^ |

Different groups of data were analyzed using one-way ANOVA based on Duncan's test to compare their significant differences. And data was represented the means (±SEM), the different letters on each line represent significant differences in different groups.

**Supplementary Table 6.** The predominant gut bacteria at genus level in zebrafish among three group.

| Genus | LFD | HFD | Amuc |
| --- | --- | --- | --- |
| *Mycobacterium* | 24.12±21.70^a^ | 28.28±14.73^a^ | 27.46±23.02^a^ |
| *Acinetobacter* | 21.41±11.68^a^ | 18.27±10.58^a^ | 3.71±1.94^b^ |
| *Vibrionaceae* | 0.49±0.67^a^ | 6.08±10.44^a^ | 20.60±27.78^a^ |
| *Staphylococcus* | 0.56±0.78^a^ | 0.40±0.48^a^ | 26.66±17.16^b^ |
| *Perlucidibaca* | 11.81±7.30^a^ | 9.27±6.63^a^ | 0.99±0.48^b^ |
| *Lactococcus* | 10.52±8.94^a^ | 6.71±4.29^ab^ | 1.61±0.98^b^ |
| *Aeromonas* | 3.95±3.31^a^ | 4.87±6.27^a^ | 2.36±2.90^a^ |
| *Pseudomonas* | 0.73±0.39^a^ | 1.25±1.09^a^ | 2.89±4.47^a^ |
| *Bacillus* | 0.0065±0.008^a^ | 0.026±0.054^a^ | 0.013±0.003^a^ |

Different groups of data were analyzed using one-way ANOVA based on Duncan's test to compare their significant differences. And data was represented the means (± SEM), the different letters on each line represent significant differences in different groups.
